# Could we employ the queueing theory to improve efficiency during future mass causality incidents?

**DOI:** 10.1186/s13049-019-0620-8

**Published:** 2019-04-11

**Authors:** Chih-Chuan Lin, Chin-Chieh Wu, Chi-Dan Chen, Kuan-Fu Chen

**Affiliations:** 1 Department of Emergency Medicine, Chang Gung Memorial Hospital, Linkou, Taiwan; 2 Department of Emergency Medicine, Chang Gung Memorial Hospital, Keelung, Taiwan; 3 Clinical Informatics and Medical Statistics Research Center, Chang Gung University, Taoyuan, Taiwan; 4 Community Medicine Research Center, Chang Gung Memorial Hospital, 5 Fu-Shin Street, Gueishan Village, Keelung, Taoyuan Taiwan 333

**Keywords:** Queueing network, Mass causality incidents, Emergency department

## Abstract

**Background:**

Preparation for a disaster or accident-related mass casualty events is often based on experience. The objective measures or tools for evaluating decision-making and effectiveness during such events are underdeveloped. Queueing theory has been suggested to evaluate the effectiveness of mass causality incidents (MCI) plans.

**Objective:**

Using different types of real MCI, we aimed to determine if a queueing network model could be used as a tool to assist in preparing plans to address mass causality incidents.

**Methods:**

We collected information from two types of mass casualty events: a motor vehicle accident and a dust explosion. Patient characteristics, time intervals of every working station, numbers of physicians and nurses attending, and time required by physicians and nurses during these two MCIs were collected and used for calculation in a queueing network model. Balanced efficiency was determined by calculating the numbers of server, i.e., nurses and physicians, in the two MCIs.

**Results:**

Efficient patient flows were found in both MCIs. However, excessive medical manpower supply was revealed when the queueing network model was applied to assess the MCIs. The best fitting result, i.e., the most efficient man power utilization, can be calculated by the queueing network models. Furthermore, balanced efficiency may be a more suitable condition than the highest efficiency man power utilization when faced with MCIs.

**Conclusion:**

The queueing network model is a flexible tool that could be used in different types of MCIs to observe the degree of efficiency when handling MCIs.

**Electronic supplementary material:**

The online version of this article (10.1186/s13049-019-0620-8) contains supplementary material, which is available to authorized users.

## Introduction

Hospitals should have a comprehensive mass-casualty incidents (MCI) preparedness plan that can be used to cope with surges in demand for healthcare. An important concept that should be included in an MCI preparedness plan is that of balance between the demand (e.g. patients) and supply (e.g., resources). Two concepts have been used to describe the aforementioned balance: surge capacity, which is traditionally defined as the maximum available resource of a health care system to meet the increased demand that occurs during an MCI [1], and surge response capability (SRC), which is the ability of the surge capacity to accommodate the surge [1]. While saving excessively on SRC during disaster events may strain ordinary daily medical needs and/or increase the workload of medical personnel, reserving too little SRC could present other issues that may put medical staff and patients in danger. Thus, effective decision-making is essential to the success of an MCI preparedness plan. The Task Force on Quality Control in Disaster Medicine of the World Association for Disaster and Emergency Medicine defined effectiveness as a quality which “relates to how closely the output matches the specified goal.” [2] Thus, establishing flexible and objective measures with which to determine effectiveness in responding to MCIs is a worthwhile endeavor.

Queueing theory (QT) has been suggested to evaluate the effectiveness of MCI plans [3]. This concept has been widely used to develop emergency department (ED) flow models to cope with ED crowding [4–6], but is rarely applied to MCIs. The theory, which includes assumptions on the probabilistic nature of the arrival at the queue, waiting in the queue, and serving the front of the queue, is a mathematical description of a queueing system. Based on QT theory, several measures of performance, including average waiting time in the queue or system, expected number waiting for or receiving service, and probability of encountering the system in certain states, could be derived. Application of this theory necessitates some trade-offs between the cost of service (providing health care) and the cost associated with waiting for that service (treatment delay). The ultimate goal of the concept is to achieve a balance between service in disaster events and ordinary clinical care. Therefore, queueing models may be used to make decisions that balance health care service (SRC) with MCI demand (surge capacity). QT usually contains only one node or station and can only manage uni-directional flow. Thus, the queueing network theory (QNT) was developed to solve the issue related to multiple stations.

Valuable lessons regarding decision-making and effectiveness have been learned through exercises and simulations [7, 8]. However, actual disaster experiences may add expert consensus regarding the meaning of optimal decision making and the effectiveness mean [2]. To the best of our knowledge, objective measures or tools to evaluate disaster management plans remain underdeveloped. We believe that applying a queueing network to different types of actual MCIs can help evaluate decision-making and effectiveness (optimal SRC) in response to MCIs. Accordingly, we aimed to derive a novel QT-based network by using real MCIs in the ED, to support decision-making in disaster management.

## Methods and materials

### MCI cases and analysis

We retrospectively collected data from two MCIs: a bus accident that occurred on a freeway in December 2013 and subsequently resulted in 41 casualties (Event A), and a dust explosion in a water park that occurred on June 2015 and subsequently resulted in 499 casualties (Event B). All of the victims in event A were sent to the Chang Gung Memorial Hospital (CGMH) in Linkou, which was a 5-min drive from the accident. In event B, forty-nine victims with severe burns were sent to CGMH for further care as the hospital is a 30-min drive from the location of the dust explosion on an ordinary day. CGMH is a tertiary academic teaching hospital with 3700 beds, serving approximately 180,000 patients in its ED annually; it is categorized as an advanced emergency response hospital by the Ministry of Health and Welfare in Taiwan [9]. The five-level validated Taiwan triage and acuity scale was implemented since 2010 in Taiwan. For the MCI and disaster planning purpose, we simplified the triage into emergency (level one and two) and urgency (other levels). We followed the Strengthening the Reporting of Observational Studies in Epidemiology (STROBE) statement to report this observational study. The institutional review board approved this study (201700662B0) and waived the informed consents owing to the retrospective nature of this study.

**Fig. 1:**
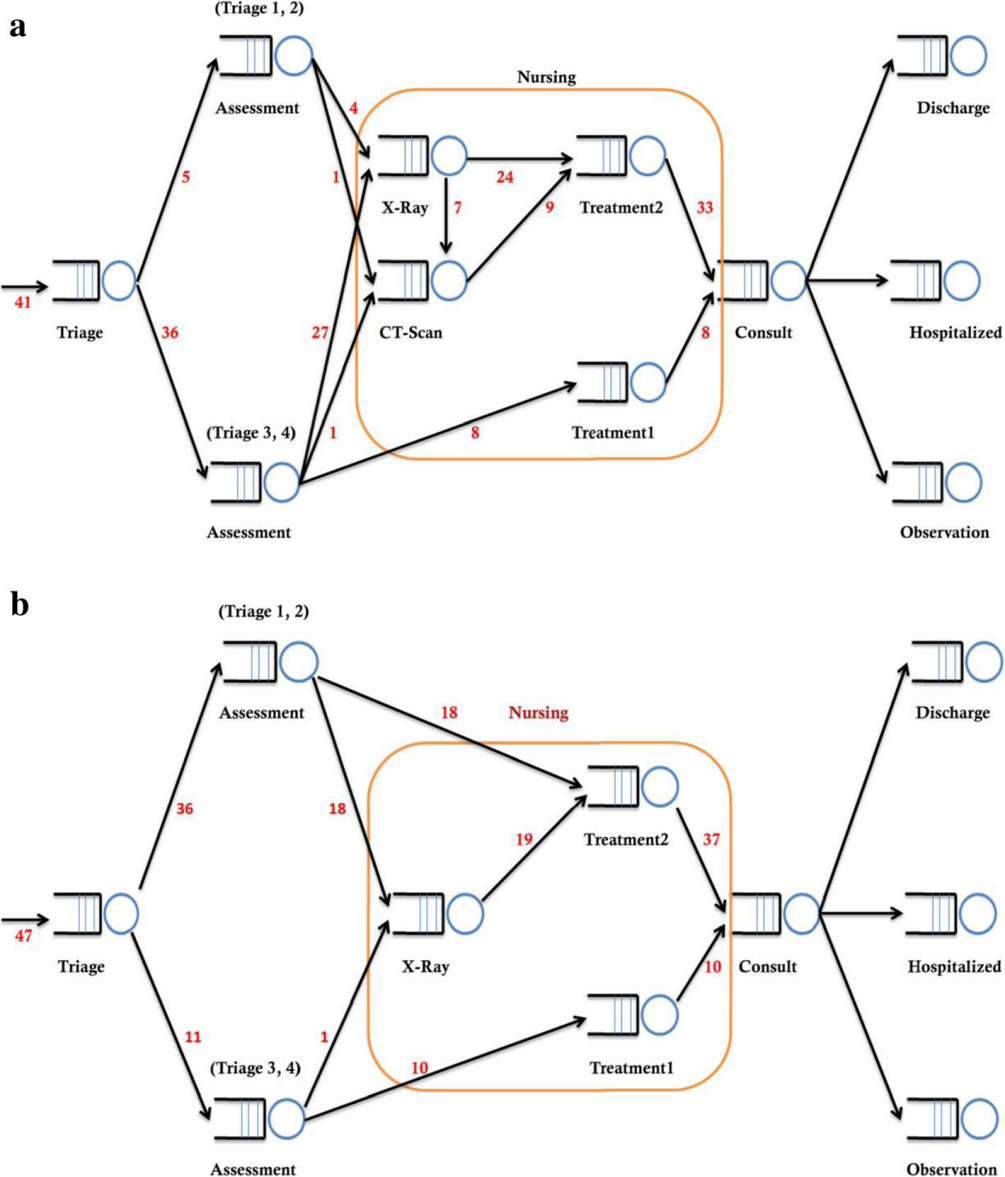
a and b patient flow during event A and event B, respectively. Triage: triage station; Triage 1,2/ 3,4 assessment: ED physicians evaluate triage 1,2 /3,4 patients; X- Ray and CT-Scan: radiology examination; Treatment 1/2: nurses execute medical orders of triage 1,2 /3,4 patients. The number in figures imply the number of patients

Figure 1a and b illustrate patient flows from one working station to another in event A and B, respectively. Patient characteristics and time intervals of every working station were obtained from electronic medical records in CGMH. For example, the times from the accident scene to the hospital, from triage to consultation with physicians, from prescription to execution, and from order execution to disposition were collected and calculated. The triage levels of patients and numbers of physicians and nurses attending during the MCIs were also recorded. The average times required for physicians to complete patient consultations, nurses to perform triage assessment, and radiology technicians to perform radiology examinations were recorded and used for further calculations in the model.

### Queueing network model

In this study, the queueing network can be considered as a system composed of an arbitrary but finite number of interconnected queues, such as patient flows. Such a network can be modeled by a set of treatment centers where each treatment center may contain one or more health-care providers. Patients traveling through the network were served at the treatment centers. Queueing networks can be further divided as open, closed, or mixed according to the different patterns in which patients enter or leave the system. In the open queueing network, patients enter the network from outside, receive treatment at one or more centers, and then leave the network. By comparison, in the closed queueing network, patients never leave or enter the network [10, 11]. The mixed queueing network is open to some patients and closed to others [12]. Furthermore, in our network queueing model, the nurses are responsible for triage and treatment stations, and the physicians were responsible for the assessment and consultation stations.

### Underlying assumptions

In this study, we focus on open queueing networks to evaluate ED operations. The following assumptions must be fulfilled for an open queueing network:

1. Each treatment center follows a first-in/ first-out queue.
2. The treatment time of queue *i* follows an exponential distribution with parameter *µ*_*i.*_
3. Upon departure from the queue *i*, the patient transfers to the next queue *j* with a probability of *q*_*i,j*_.
4. The network is open to arrivals from outside the network, where the source *s* patients arrive as a Poisson distribution with a mean arrival rate *λ* and a fraction *q*_*s,i*_ of these enter the queue *i* with intensity *λ* *q*_*s,i*_.

### Performance

The measures of performance for a waiting-line system in the queueing analysis can be derived based on two statistics: the mean arrival rate (λ) and the mean treated rate (µ). The following performance indicators were used in the analysis [13].

1. *ρ*: Utilization factor for the system (what proportion of time the treatment center is busy) or efficiency, the higher this factor, the more the system is utilized. In MCI setting, balance between under or over-efficient is important since the resource is limited. (*ρ* = *λ* / *µ*)
2. L_s_: Average number of patients in the system, including patients who are being treated and who are waiting to be treated, similar to the concept of crowding. (L_s_ = *ρ* / (1 – *ρ*) = *λ* / (*µ* – *λ*))
3. L_q_: Average length of the queue or the average number of patients in a line awaiting service. (L_q_ = L_s_ - *ρ* = *ρ*^2^ / (1 – *ρ*))
4. W_s_: Average time a patient stays in the system (waiting time plus treatment time). (W_s_ = L_s_ / *λ* = 1 / (*µ* – *λ*))
5. W_q_: Average waiting time or the average length of time a patient waits before being treated. (W_q_ = L_q_ / *λ* = *ρ* / (*µ* – *λ*))

The statistics of the arrival rate (*λ*) and the treated rate (*µ*) were also determined from the available data using the observed average for each station, but with some adjustments according to consensus meetings. The open queueing network analysis was performed using Queueing Theory Software (QTS) [13]. Furthermore, in order to test the sensitivity of influence of insufficiency of health care provider called to the ED to support from the institute, we conducted a sensitivity analysis using half the mean treated rate (*µ*) to test the performance.

## Results

Table 1 lists the characteristics of the patients in events A and B; the median ages of the patients were 27 years (range, 20–39 years) and 20 years (range, 19–24 years), respectively. While 65.9% of the patients in event A were triaged with triage as urgency (level three acuity), 76.6% of the patients in event B were triaged as emergency (level one or two acuity). Considering this difference in triage acuity, the two events may be considered to be quite different from each other.

**Table 1:** Patient Characteristics in event A and event B

	Event A (*n* = 41)	Event B (*n* = 47)
Age, year, Median (Range)	27 (20–39)	20 (19–24)
Gender, No. (%)		
Male	14 (34.15%)	20 (42.55%)
Female	27 (65.85%)	27 (57.45%)
Triage, No. (%)		
1	1 (2.44%)	32 (68.09%)
2	4 (9.76%)	4 (8.51%)
3	27 (65.85%)	11 (23.4%)
4	9 (21.95%)	0 (0%)

Patients began arriving at the CGMH ED about 20 min after the accident in event A. The last patient arrived 70 min after the accident. Thus, for event A, a rapid surge in patient admission occurred within 50 min. In contrast, for event B, the hospital began to receive patients 19 min after the dust explosion, and the last patient arrived 160 min after the accident (Additional file 1: Figure S1). The calculated average of events A and B were 41 and 22 persons per hour, respectively. Severe traffic jams occurred near the dust explosion location, and retrieval of the victims was challenging because of the remote location of the accident. These two factors could have influenced the slow *λ* of patients at the hospital in event B. When event B occurred, news outlets estimated nearly 500 victims. Thus, a large number of ED physicians, trauma surgeons, and nurses were called back to CGMH to prepare for the incoming surge of patients from event B. Three hundred and thirty-six staff members, including 90 doctors (19 plastic surgeons, 11 ED doctors, and 8 trauma surgeons), 184 nurses, 21 administrators, and 41 paramedical staff were called to various hospitals that night. Five patients with superficial secondary burns on less than 5% of their total body surface area were discharged directly from the ED. Seven patients with mild burn injuries were admitted to ordinary wards, 25 patients were admitted to the burn unit, and 12 patients were admitted to the microsurgical intensive care unit [14]. The mean ED lengths of stay for events A and B were 88.74 ± 35.39 and 141.74 ± 113.70 min, respectively.

Table 2 shows the service intervals of events A and B. Execution of medical orders by the nurses was the most time-consuming service. Using the service intervals listed in Table 2, the results of the queueing network for events A and B (Fig. 1) were subsequently calculated and are shown in Tables 3 and 4. For event A and B, the mean arrival rates *λ* at the triage station were 41 and 22 patients per hour and mean treated rates *µ* were 15 and 6 patients per hour, respectively. Given three servers in the triage station, the average number of people in the system (L_s_) were 11.3 and 12.7, and the average number of people in a line awaiting service (L_q_) were 8.58 and 9.04, respectively. The average time for a customer in the system (waiting time plus service time; W_s_) were 0.28 and 0.58 h, while the average waiting time (W_q_) were 0.21 and 0.41 h, respectively. The efficiency (*ρ*) were 91.11 and 91.67%, respectively. When the number of servers was increased to 4 or 5 per triage station in event A, the efficiency (*ρ*) was decreased to 68.3 and 54.7%, respectively. We also illustrate how insufficiency of health care provider can influence the estimated numbers of servers in the Additional file 2: Table S1.

**Table 2:** Service Intervals of Events A and B

		Event A				Event B			
Service Intervals (minutes)	Triage category	No. (%)	Mean ± SD	Min	Max	No. (%)	Mean ± SD	Min	Max
Time form accident happened to ED admission time	All	41(100)	48.07 ± 12.48	23	65	47 (100)	64.35 ± 41.00	18	153
Time form ED admission time to triage	All	41(100)	3.76 ± 1.45	1	7	47 (100)	10.45 ± 10.12	1	42
Time form triage to ED physician evaluate patient	Emergency	5 (12.2)	11 ± 4.64	7	17	36 (76.6)	13.75 ± 9.24	1	36
	Urgency	36 (87.8)	12.86 ± 4.90	2	22	11 (23.4)	8.55 ± 4.59	4	18
Time of nurse execute the medical orders	Emergency	5 (12.2)	82.6 ± 33.02	35	117	36 (76.6)	150.97 ± 111.61	8	470
	Urgency	36 (87.8)	71.52 ± 30.48	28	144	11 (23.4)	88 ± 120.32	6	338
Time form X ray ordered to patient been sent to X ray room	All	31(75.61)	1.45 ± 0.87	1	4	27 (57.45)	10.58 ± 10.44	1	35
Time for taking X ray	All	31(75.61)	12.31 ± 5.73	2	22	27 (57.45)	12.23 ± 9.22	1	29
Time form CT scan ordered to patient been sent to T scan room	All	9(21.95)	1.75 ± 1.16	1	4	–	–	–	–
Time for taking CT scan	All	9(21.95)	20.33 ± 15.40	4	48	–	–	–	–
ED stay length from triage to discharge	Triage 1 and 2	5 (12.2)	87.67 ± 33.84	59	125	36 (76.6)	147.05 ± 103.37	8	407
	Triage 3 and 4	36 (87.8)	84.81 ± 36.83	16	158	11 (23.4)	79.67 ± 117.22	1	339

*For MCI planning purposes: emergency was defined as triage level one and two, urgency defined as other triage levels

**Table 3:** The results of queueing network for events A

Queue Station^b^	Triage			Assessment12^a^			Assessment34^a^			Treatment1			Treatment2			Consult		
*λ*	41			5.002			35.998			7.99			33.008			41		
*μ*	15			4			6			6			6			12		
Servers	3	4	5	2	3	4	7	8	9	2	3	4	6	7	8	4	5	6
*L*_*s*_	11.310	3.603	2.945	2.053	1.362	1.269	9.681	7.070	6.391	2.393	1.476	1.358	14.124	7.179	6.055	7.501	4.176	3.632
*L*_*q*_	8.577	0.870	0.211	0.803	0.111	0.019	3.681	1.070	0.392	1.062	0.144	0.026	8.623	1.677	0.554	4.084	0.759	0.215
*W*_*s*_ (hour)	0.276	0.088	0.072	0.410	0.272	0.254	0.270	0.196	0.178	0.299	0.185	0.170	0.428	0.217	0.183	0.183	0.102	0.089
*W*_*q*_ (hour)	0.2091	0.021	0.005	0.160	0.022	0.004	0.102	0.029	0.011	0.133	0.018	0.003	0.261	0.051	0.017	0.100	0.019	0.005
*ρ* (%)	91.11	68.33	54.67	62.53	41.68	31.26	85.71	75.00	66.66	66.60	44.40	33.30	91.69	78.59	68.77	85.42	68.33	56.94

^a^Assess12: assessment for triage 1 and 2. Assess34: assessment for triage 3 and 4

^b^*λ* the arrival rate, *µ* the served rate, *L*_*s*_ Average number of people in the system, *L*_*q*_ Average length of the queue or the average number of people in a line awaiting service, *W*_*s*_ Average time for a customer in the system (waiting time plus service time), *W*_*q*_ Average waiting time or the average length of time that a customer waits before being served, *ρ* utilization factor for the system

**Table 4:** The results of queueing network for events B

Queue Station^b^	Triage			Assessment12^a^			Assessment34^a^			Treatment1			Treatment2			Consult		
*λ*	22			16.852			5.148			4.6795			17.3205			22		
*µ*	6			6			12			2			1			12		
Servers	4	5	6	3	4	5	1	2	3	3	4	5	18	19	20	2	3	4
L_s_	12.706	4.857	3.997	15.755	3.827	3.054	0.751	0.449	0.431	4.519	2.718	2.431	38.249	23.545	20.126	11.478	2.413	1.948
L_q_	9.039	1.190	0.330	12.946	1.018	0.245	0.322	0.021	0.002	2.179	0.378	0.091	20.928	6.225	2.805	9.645	0.580	0.115
W_s_ (hour)	0.578	0.221	0.182	0.935	0.227	0.181	0.146	0.087	0.084	0.966	0.581	0.519	2.208	1.359	1.162	0.522	0.109	0.088
W_q_ (hour)	0.411	0.054	0.015	0.768	0.060	0.015	0.063	0.004	0.0003	0.466	0.081	0.019	1.208	0.359	0.162	0.438	0.026	0.005
*ρ* (%)	91.67	73.33	61.11	93.62	70.22	56.17	42.90	21.45	14.30	77.99	58.49	46.80	96.22	91.16	86.60	91.67	61.11	45.83

^a^Assess12: assessment for triage 1 and 2. Assess34: assessment for triage 3 and 4

^b^*λ* the arrival rate, *µ* the served rate, *L*_*s*_ Average number of people in the system, *L*_*q*_ Average length of the queue or the average number of people in a line awaiting service, *W*_*s*_ Average time for a customer in the system (waiting time plus service time), *W*_*q*_ Average waiting time or the average length of time that a customer waits before being served, *ρ* utilization factor for the system

The most efficient manpower utilization rates (the best fitting results, the highest *ρ*) of the queueing network for events A and B can be obtained through our model (Table 5). The best fitting model predicted the need for three nurses at the triage station, two ED physicians to evaluate the emergency (triage one and two) patients, seven ED physicians to evaluate the urgent (triage level three and four) patients, two nurses to treat less severe patients and six nurses to treat the more severe patients in event A (Table 5). Therefore, nine ED physicians and 11 nurses were actually necessary. However, only three ED physicians but 30 nurses were involved in event A. In event B, the best fitting model indicated the need for four nurses in the triage station, three ED physicians to evaluate the more severe patients, one ED physician to evaluate the less severe patients, three nurses to treat the less severe patients and 18 nurses in triage to treat the more severe patients. Therefore, the model predicted a need for only four ED physicians and 25 nurses. However, 15 ED physicians and 120 nurses were involved in event B.

**Table 5:** The best fitting results of queueing network for Events A and B

Event	A								B						
Queue Station^b^	Triage	Assess12^a^	Assess34^a^	X-ray	CT^a^	Tre1^a^	Tre2^a^	Consult	Triage	Assess12^a^	Assess34^a^	X-ray	Tre1^a^	Tre2^a^	Consult
*λ*	41	5	35.99	31	9.01	7.99	33.01	41	22	16.85	5.15	8.89	4.68	17.32	22
*μ*	15	4	6	20	10	6	6	12	6	6	12	20	2	1	12
Servers	3	2	7	2	1	2	6	4	4	3	1	1	3	18	2
*L*_*s*_	11.31	2.05	9.68	3.88	9.15	2.39	14.12	7.50	12.71	15.75	0.75	0.80	4.52	38.25	11.48
*L*_*q*_	8.58	0.80	3.68	2.33	8.24	1.06	8.62	4.08	9.04	12.95	0.32	0.36	2.18	20.93	9.64
*W*_*s*_ (hour)	0.28	0.41	0.27	0.13	1.01	0.30	0.43	0.18	0.58	0.93	0.15	0.09	0.97	2.21	0.52
*W*_*q*_ (hour)	0.21	0.16	0.10	0.07	0.91	0.13	0.26	0.10	0.41	0.77	0.06	0.04	0.47	1.21	0.44
*ρ* (%)	91.11	62.53	85.71	77.50	90.14	66.60	91.69	85.42	91.67	93.62	42.90	44.47	77.79	96.22	91.67

^a^Assess12: assessment for triage 1 and 2. Assess34: assessment for triage 3 and 4. *CT* computed tomography scan. Tre1: Treatment 1. Tre2: Treatment 2

^b^*λ* the arrival rate, *µ* the served rate, *L*_*s*_ Average number of people in the system, *L*_*q*_ Average length of the queue or the average number of people in a line awaiting service, *W*_*s*_ Average time for a customer in the system (waiting time plus service time), *W*_*q*_ Average waiting time or the average length of time that a customer waits before being served, *ρ* utilization factor for the system

## Discussion

In this study, we utilize QT as mathematical models to allow system designers to calculate performance metrics (such as average queue length, average wait time, and the proportion of customers turned away) in health care operations [15]. Previous studies mainly focus on the emergency cardiac ward, intensive care unit, entire hospital, or general operational research settings [16–21]. In this study, we applied queueing network to different kinds of MCIs to examine the degree of efficiency when handling these events. Using these models, we demonstrated how to use QT in evaluating patient arrival, efficient patient flow, length of stay, efficient patient outflow and physician and nursing manpower in two different kinds of MCIs. We believe that this study is the first to apply QT to evaluate the decision-making and effectiveness (optimal SRC) of different types of MCI management plans in the ED.

### Patient arrival, efficient patient flow and length of stay

Creating an efficient patient flow is key to successfully cope with a surge during an MCI. To achieve efficient patient flow, high efficiency with high SRC is necessary at every working station in the ED during MCIs. The characteristics of an efficient patient flow include high patient throughput, a short length of stay (W_s_ and W_q_), maintenance of a sufficient resource utilization rate (*ρ*), and low staff idle time [22]. The length of stay in an ED during an MCI could serve as an index of efficiency in managing MCIs. In this study, we found that patients from event B had a much longer length of stay in the ED than those from event A. This finding could be explained by differences in the type of MCI, the arrival rate of triage (*λ*), the severity of patient condition (time needed to evaluate and treat patients), and the output time interval between events, all of which may influence the length of stay in the ED.

An important modifiable factor is the arrival rate, which has an ongoing effect on a patient’s length of stay as a function of its impact on waiting time [23]. Event A had a higher arrival rate (λ) than event B in triage; however, a shorter length of stay in the ED was observed in event A. Event B was an MCI characterized by a large number of severe burn injury patients, while event A was a traffic accident with patients who suffered only mild injuries. As the type of MCI may determine the severity of patients’ conditions, event B required longer lengths of stay in the ED than event A. Using the queueing network, much longer time patients staying the system (W_s_) and waiting to be treated (W_q_) and fewer patients in our triage and ED (L_s_ and L_q_) can be observed in the more severe patients in event B but not in event A.

A queueing network could be used in different types of MCIs to examine the degree of efficiency when handing these events. In the two MCI cases we studied, short length of stay (W_s_ and W_q_), sufficient resource utilization (*ρ*) and the absence of staff idle time (determined via discussion with the head nurses participating in the two MCIs) were observed. A shortage of manpower during MCIs may cause high levels of stress and an excessive workload for physicians, even when patient injuries are less serious. In event A, three ED physicians and 30 nurses were available. However, according to the queueing network, the availability of up to nine ED physicians and only 11 nurses was considered to be the most efficient strategy (the highest ρ in each working station) to cope with event A. In contrast, excessive availability of medical staff in event B was observed when the queueing network was applied to examine it. Specifically, 90 physicians and 184 nurses were available in event B. However, according to the best fitting result of queueing network, only four physicians and 25 nurses were needed. Excess staff availability during MCI is a pitfall in managing MCIs. In event A, an excessive number of nurses and inadequate physician manpower were observed in this study. Thus, the queueing network could be used as a tool to analyze the factors influencing the length of stay in the ED in different types of MCIs.

### Applying queueing network to estimate manpower and to reach balanced efficiency

In addition to the balance between surge capacity and SRC, the balance between urgency and efficiency should be considered when managing MCIs in the ED. Studies on fast track intervention for less urgent patients [24] showed that reducing waiting times could achieve high efficiency [25]. The queueing model showed us that seven servers (physicians) should be activated as the most efficient in managing patients in event A (working treatment station one and two, i.e., patients of triage three and four, 88% of all victims). Thus, the addition of more servers, in this case, physicians, as suggested by the queueing model, could ensure that efficiency is guaranteed in the urgent situation.

In addition to physician manpower, nursing plays a critical role in determining hospital costs care quality, and patient satisfaction [26]. The minimum nurse-to-patient ratio is the most commonly used method to determine nurse staffing levels [27]. Queueing models, such as which is presented in the present study, can flexibly capture the stochastic nature of surge capacity during an MCI; therefore, it may be a good tool for determining nurse staffing levels during emergency events. If λ can be precisely estimated, queueing models can help a hospital manager to determine how many and to what extent medical staff should be alerted.

When a queueing network is applied, the general objective is to achieve maximum efficiency in a working system. However, some lessons can be learned from the excessive availability of medical staff in event B. More servers (e.g., doctors, nurses.) were available than were required to achieve the maximum efficiency calculated by the queueing network. Although the efficiency of each server is decreased when more servers are added, however, the participating servers may feel less stress, their workloads may lighten, and efficient patient flow is still achieved. Furthermore, health care providers pulled from other departments into ED could hazard the patient safety as well. Therefore, “balanced efficiency” could be a better approach than maximum efficiency to cope with surges during MCIs. Using a queueing network, the most efficient working situation can be calculated. Since the model is a flexible tool, the number of servers can be increased to reduce stress and workloads without marked losses in efficiency.

### Applying queueing network to cope with MCIs in different EDs

In event B, the lengths of stay in the ED were influenced by the output time intervals. In this event, the median output time (time interval between the disposition decision and patient discharge) was 56 min (15.3–117.3, IQR) [22]. Such a short output time does not delay patient admission and higher efficiency can be achieved by providing fewer servers, as predicted by QT. As stated in the 2006 Academic Emergency Medicine Consensus Conference, “Key leadership for surge capacity planning and response varies by locality and is generally not well defined.” The same conference found that “no studies have suggested how leadership should be adapted in response to various types of events.” [28] Therefore, a queueing network may be a useful tool in coping with different types of MCIs in different EDs for SRC estimation.

We suggest that the queueing network could be used in the planning section of the hospital emergency incident command system (HEICS). The functions of the planning section are to collect, evaluate, and disseminate incident situation information and intelligence to Incident command. Collecting the queuing network variables in historical MCI cases could be a scientific mean in hospital emergency management programs. The planning chief could develop an action plan for operation by using queueing network during a disaster incident.

### Limitations

One oversimplified limitations for which the queueing model is often criticized is its assumptions on working time in each station [29]. These assumptions must be considered according to the staffing availability of individual hospitals. Therefore, the numbers related to how many medical staff should be alerted in the present cannot be directly applied to other hospitals. However, this limitation does not interfere with the applicability of the queueing network model to different MCIs or hospitals. A second limitation is that this study is a retrospective study in the ED; therefore; the data retrieval process may be less precise than what would be ideal. Building an accurate queueing model for ED systems in coping with MCIs is challenging. Despite the care taken when extracting records from the electronic data base, data of two victims from event B were clearly inaccurate, and we have to exclude these patients from this study. Incomplete or inaccurate records, and unavailable or censored data may be obtained in the rush to address MCIs. The above limitations may result in unrealistic data inputs, thereby influencing modeling accuracy and the validity of our results. Therefore, caution should be observed when applying these results. Third, nonhuman resource management was not included or discussed in this study. Fortunately, as the SRC of CGMH is fairly large, so this lack of consideration of nonhuman resources does not influence the outcomes of our queueing network analysis [29]. Fourth, the lack of data of patient outcome and cost of the increase of manpower limit the detailed cost-effectiveness comparison in this study. Fifth, we have no outflow data such as time to OR or ICU in our dataset, which could serve as the bottlenecks during the MCIs. Sixty, we do not have any detailed severity scores that could divide patients into more detailed severity stratum. Lastly, this study was conducted in a tertiary teaching hospital. The Chang Gung Memorial Hospital in Linkou (CGMH) is a 3700-bed hospital with 12,000 monthly ED visits, and much of its reserved SRC cannot be employed in other smaller hospitals. These reserved surge resources, including rapid admission of patients to the suitable wards, would diminish the ongoing effect of arrival rate on a patient’s length of stay. Further studies are merited for the generalizability.

## Conclusions

A queueing network is a flexible and efficient tool that can be used in different MCIs to confirm the balance between surge capacity and SRC. It can also be applied to improve balanced efficiency when coping with MCIs. Balanced efficiency may be more advantageous than the best fitted results, i.e. the most efficiency that was calculated by the queueing network. Efficiency and appropriate human resources can be considered as coexisting in the most ideal situation when a queueing network is applied.

## Additional files

Additional file 1: **Figure S1.** Arrival time table and distribution of event A and B. (PDF 292 kb)

Additional file 2: **Table S1a.** The results of sensitivity analysis for insufficient health care providers in queueing network for events A. **Table S1b.** The results of sensitivity analysis for insufficient health care providers in queueing network for events B. (DOCX 44 kb)
