# Supplementary figures and images for: Could we employ the queueing theory to improve efficiency during future mass causality incidents?
Source: Scand J Trauma Resusc Emerg Med. 2019 Apr 11;27:41. doi: 10.1186/s13049-019-0620-8 (PMC6458797; doi:10.1186/s13049-019-0620-8)

Supplement figure: Arrival time table and distribution of event A and B

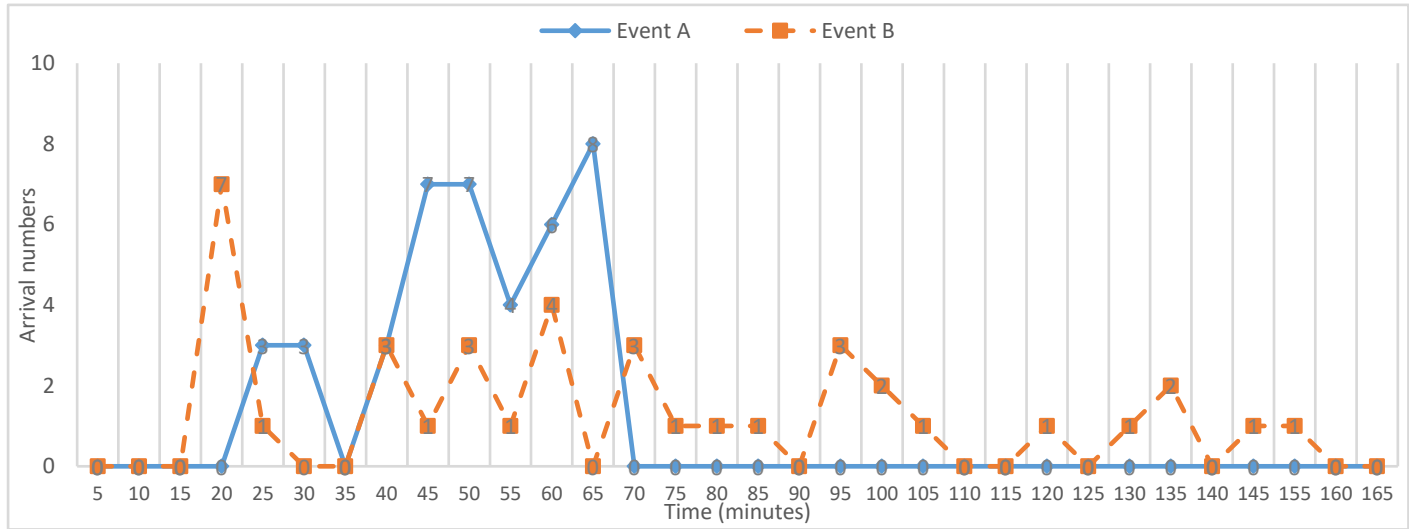

Supplement: Supplementary file 1 — Figure S1. Arrival time table and distribution of event A and B. (PDF 292 kb) [file 13049_2019_620_MOESM1_ESM.pdf]
