# Supplementary material for: Could we employ the queueing theory to improve efficiency during future mass causality incidents?
Source: Scand J Trauma Resusc Emerg Med. 2019 Apr 11;27:41. doi: 10.1186/s13049-019-0620-8 (PMC6458797; doi:10.1186/s13049-019-0620-8)
Supplement: Supplementary file 2 — Table S1a. The results of sensitivity analysis for insufficient health care providers in queueing network for events A. Table S1b. The results of sensitivity analysis for insufficient health care providers in queueing network for events B. (DOCX 44 kb) [file 13049_2019_620_MOESM2_ESM.docx]

**Additional file 2: Table S1a: The results of sensitivity analysis for insufficient health care providers in queueing network for events A.**

| Queue Station | Triage | | | Assessment12 | | | Assessment34 | | | | | Treatment1 | | | Treatment2 | | | | | Consult | | |
| --- | --- | --- | --- | --- | --- | --- | --- | --- | --- | --- | --- | --- | --- | --- | --- | --- | --- | --- | --- | --- | --- | --- |
| $\lambda$ | 41 | | | 5.002 | | | 35.998 | | | | | 7.99 | | | 33.008 | | | | | 41 | | |
| $\mu$ | 15 | | | 2 | | | 3 | | | | | 3 | | | 3 | | | | | 12 | | |
| Servers | 3 | 4 | 5 | 3 | 4 | 5 | | 12 | 13 | 14 | 3 | | 4 | 5 | | 12 | 13 | 14 | 4 | | 5 | 6 |
| $L_{s}$ | 11.310 | 3.603 | 2.945 | 8.957 | 3.415 | 2.847 | | 18.666 | 13.574 | 12.116 | 3 | | 3.415 | 2.847 | | 18.666 | 13.574 | 12.116 | 7.501 | | 4.176 | 3.632 |
| $L_{q}$ | 8.577 | 0.870 | 0.211 | 6.294 | 0.752 | 0.183 | | 7.663 | 2.572 | 1.113 | 8.957 | | 0.752 | 0.183 | | 7.663 | 2.572 | 1.113 | 4.084 | | 0.759 | 0.215 |
| $W_{s}$ (hour) | 0.276 | 0.088 | 0.072 | 1.121 | 0.427 | 0.356 | | 0.565 | 0.411 | 0.367 | 6.294 | | 0.427 | 0.356 | | 0.565 | 0.411 | 0.367 | 0.183 | | 0.102 | 0.089 |
| $W_{q}$ (hour) | 0.2091 | 0.021 | 0.005 | 0.788 | 0.094 | 0.023 | | 0.232 | 0.099 | 0.034 | 1.121 | | 0.094 | 0.023 | | 0.232 | 0.099 | 0.034 | 0.100 | | 0.019 | 0.005 |
| $\rho$ (%) | 91.11 | 68.33 | 54.67 | 88.78 | 66.58 | 53.27 | | 91.69 | 85.42 | 78.59 | 0.788 | | 66.58 | 53.27 | | 91.69 | 85.42 | 78.59 | 85.42 | | 68.33 | 56.94 |

**Additional file 2: Table S1b: The results of sensitivity analysis for insufficient health care providers in queueing network for events B.**

| Queue Station | Triage | | | Assessment12 | | | Assessment34 | | | | | Treatment1 | | | Treatment2 | | | | | Consult | | |
| --- | --- | --- | --- | --- | --- | --- | --- | --- | --- | --- | --- | --- | --- | --- | --- | --- | --- | --- | --- | --- | --- | --- |
| $\lambda$ | 22 | | | 16.852 | | | 5.148 | | | | | 4.6795 | | | 17.3205 | | | | | 22 | | |
| $\mu$ | 6 | | | 3 | | | 6 | | | | | 1 | | | 1 | | | | | 12 | | |
| Servers | 4 | 5 | 6 | 5 | 6 | 7 | | 4 | 5 | 6 | 5 | | 6 | 7 | | 4 | 5 | 6 | 5 | | 6 | 7 |
| $L_{s}$ | 12.706 | 4.857 | 3.997 | 17.011 | 6.373 | 5.189 | | 12.706 | 4.857 | 3.997 | 17.011 | | 6.373 | 5.189 | | 12.706 | 4.857 | 3.997 | 17.011 | | 6.373 | 5.189 |
| $L_{q}$ | 9.039 | 1.190 | 0.330 | 12.331 | 1.694 | 0.510 | | 9.039 | 1.190 | 0.330 | 12.331 | | 1.694 | 0.510 | | 9.039 | 1.190 | 0.330 | 12.331 | | 1.694 | 0.510 |
| $W_{s}$ (hour) | 0.578 | 0.221 | 0.182 | 3.635 | 1.362 | 1.109 | | 0.578 | 0.221 | 0.182 | 3.635 | | 1.362 | 1.109 | | 0.578 | 0.221 | 0.182 | 3.635 | | 1.362 | 1.109 |
| $W_{q}$ (hour) | 0.411 | 0.054 | 0.015 | 2.635 | 0.362 | 0.109 | | 0.411 | 0.054 | 0.015 | 2.635 | | 0.362 | 0.109 | | 0.411 | 0.054 | 0.015 | 2.635 | | 0.362 | 0.109 |
| $\rho$ (%) | 91.67 | 73.33 | 61.11 | 93.59 | 77.99 | 66.85 | | 91.67 | 73.33 | 61.11 | 93.59 | | 77.99 | 66.85 | | 91.67 | 73.33 | 61.11 | 93.59 | | 77.99 | 66.85 |
